# High rates of kidney impairment among older people (≥ 60 years) living with HIV on first-line antiretroviral therapy at screening for a clinical trial in Kenya

**DOI:** 10.1371/journal.pone.0285787

**Published:** 2023-06-23

**Authors:** Jeremy Penner, Loice Achieng Ombajo, Davies Otieno, Joseph Nkuranga, Margaret Mburu, Simon Wahome, Anton Pozniak, Sanjay Bhagani

**Affiliations:** 1 Center for Epidemiological Modelling and Analysis, University of Nairobi, Nairobi, Kenya; 2 Department of Family Practice, University of British Columbia, Vancouver, Canada; 3 Department of Clinical Medicine and Therapeutics, University of Nairobi, Nairobi, Kenya; 4 Kenyatta National Hospital, Nairobi, Kenya; 5 London School of Hygiene and Tropical Medicine, London, United Kingdom; 6 Department of Infectious Diseases/HIV Medicine, Royal Free London Foundation Trust, London, United Kingdom; National Health Laboratory Service and University of Witwatersrand, SOUTH AFRICA

## Abstract

**Background:**

There is a paucity of data on kidney impairment among older people living with HIV (PLWH). We evaluated kidney function among PLWH age ≥ 60 years on first-line antiretroviral (ARV) therapy during screening for a clinical trial in Kenya.

**Methods:**

The bictegravir/emtricitabine/tenofovir alafenamide (B/F/TAF) Elderly Study is an open-label, randomized, active-controlled, non-inferiority trial conducted at two sites in Kenya. Potential participants were screened for study entry if they were at least 60 years old, had been on ARVs for at least 24 weeks and had no history of treatment failure. At screening, participants had samples collected for serum creatinine and estimated glomerular filtration rate (eGFR) was calculated using the Chronic Kidney Disease Epidemiology Collaboration 2021 equation.

**Results:**

Between January and April 2022, 714 participants were screened and had creatinine measured. All participants were black, 54.1% were female and the median age was 64 years (range 60 to 87 years). Most participants (666 [93.3%]) were on tenofovir disoproxil fumarate-containing regimens, 711 (99.6%) were on dolutegravir-containing regimens, and only 2 (0.3%) were on a regimen with a ritonavir-boosted protease inhibitor. Most participants (686 [96.6%]) were virally suppressed. Treatment for comorbidities was common, with 175 (24.5%) on treatment for hypertension and 39 (5.5%) on treatment for diabetes mellitus. The median eGFR was 64.7 mL/min/1.73m^2^, and 289 (40.5%) participants had an eGFR < 60 mL/min/1.73m^2^. In multivariate analysis, factors associated with lower eGFR were female gender (p<0.001), being on treatment for hypertension (p<0.001) and nadir CD4 count < 50 cells/μL (p = 0.008).

**Conclusions:**

Our study identified high rates of impaired kidney function among elderly PLHW in Kenya, which highlights the importance of routine assessment of kidney function and the need to address modifiable risk factors, use of appropriate ARVs, and management of kidney disease in this population.

## Introduction

Advances in antiretroviral (ARV) therapy have increased life expectancy for people living with HIV (PLWH) [1, 2] and global trends indicate that an increasing proportion of PLWH are older adults, including in sub-Saharan Africa [3, 4]. PLWH face an increased risk of non-communicable diseases associated with chronic HIV infection, toxicities from long-term use of ARVs, aging and other traditional risk factors [5, 6]. Chronic kidney disease (CKD) is a major contributor to morbidity and mortality among PLWH [7, 8], who also have a higher prevalence of CKD than the general population [9].

Long-term use of tenofovir disoproxil fumarate (TDF) and some protease inhibitors is associated with increased risk of CKD [10–12]. The kidney toxicity of TDF may be exacerbated when used in a regimen which includes ritonavir or cobicistat boosters [13]. The World Health Organization (WHO) recommends TDF-containing ARV regimens for adults and adolescents [14], and TDF is a component of the majority of regimens for PLWH on treatment in Kenya.

CKD is an important health burden among the general population in sub-Saharan Africa [15, 16], and a recent study found that using serum creatinine to estimate kidney function among adults in Malawi, South Africa and Uganda substantially underestimated the level of kidney impairment in these populations [17]. A meta-analysis found the pooled prevalence of CKD among PLWH in East Africa ranged from 5.3–20.2% when CKD was defined as estimated glomerular filtration rate (eGFR) < 60 mL/min/1.73m^2^ (using the Chronic Kidney Disease Epidemiology Collaboration (CKD-EPI) equation or Modification of Diet in Renal Disease (MDRD) study equation) or creatinine clearance (CrCl) < 60 mL/min (using the Cockcroft Gault (CG) equation); however, the median age of participants in these studies ranged from 30 to 37 years [18]. Among patients attending a clinic dedicated to people aging with HIV in the United Kingdom, with mean age of 56.5 years and 84.2% of whom were white, 7.5% were diagnosed with CKD [19]. We are not aware of published data on prevalence of kidney impairment among elderly PLWH in Kenya or the region.

In this manuscript we report the prevalence of kidney impairment among HIV-1 positive elderly adults on first-line ARV therapy receiving outpatient HIV care in Kenya, collected cross-sectionally during screening for a clinical trial.

## Materials and methods

### Study design and population

The bictegravir/emtricitabine/tenofovir alafenamide (B/F/TAF) Elderly Study is a prospective, open-label, randomized, active-controlled, multicentre, 48-week non-inferiority study describing the efficacy and safety of switching from current ARV regimen to B/F/TAF among virally suppressed older adults (≥ 60 years) in Kenya.

Study participants were recruited from outpatient HIV clinics at two public hospitals in Kenya: Kenyatta National Hospital in Nairobi, and Jaramogi Oginga Odinga Teaching and Referral Hospital in Kisumu. Participants were invited to a B/F/TAF Elderly Study screening visit if they were at least 60 years old, had been on ARV therapy for at least 24 weeks and had no history of treatment failure.

### Data collection

Demographic, clinical and laboratory characteristics were collected cross-sectionally at screening, including ARV history, medical history, and blood samples for HIV-1 RNA viral load, creatinine, aspartate aminotransferase, alanine aminotransferase, hepatitis B surface antigen, and complete blood count. Serum creatinine was quantified by the enzymatic method using an Abbott Architect CI 4100 (Abbott, Illinois, U.S.A) and eGFR was calculated with the CKD-EPI 2021 (race-neutral) equation [S1 File].

### Statistical analysis

For presentation of screening results in this manuscript, data from all participants who provided informed consent and underwent screening serum creatinine for the B/F/TAF Elderly Study were included. Baseline continuous variables were summarized using medians (interquartile ranges) except age (median, minimum, maximum), while categorical variables were summarized using counts and percentages. An adjustment for eGFR was performed to account for the predicted effect of dolutegravir (DTG) on CrCl, which Koteff et al found results in a decrease in CrCl of approximately 10% when DTG is administered at 50 mg daily [20], through inhibition of organic cation transporter 2 and was considered not clinically significant because of no reduction in true GFR [20, 21]. An eGFR < 60 mL/min/1.73m^2^ was considered the threshold for moderate impairment of kidney function. Univariate and multivariate regression analysis were performed to evaluate the association between demographic, clinical and laboratory characteristics with eGFR, reporting the p-values. All statistical tests were evaluated for significance at the 5% level. The final multivariate model excluded variables with low counts which made the model unstable. For comparison, eGFR was also estimated using the MDRD race-neutral equation and CrCl was estimated using the CG equation [S1 File] and correlation coefficients were calculated to compare with eGFR estimated using CKD-EPI 2021. All statistical analyses were performed using Stata version 15.1.

### Ethical considerations

The study was approved by the Ethics Review Committees of Kenyatta National Hospital, University of Nairobi and Jaramogi Oginga Odinga Teaching and Referral Hospital. All participants provided written informed consent. The B/F/TAF Elderly Study is registered at clinicaltrials.gov, NCT05243602.

## Results

Between January 2022 and April 2022, 718 adults provided consent for study screening, of whom screening serum creatinine was available for 714. All participants were black, all had HIV-1 infection, 386 (54.1%) were female and the median age was 64 years (range 60 to 87 years) (Table 1). Most participants (666 [93.3%]) were on TDF-containing regimens, 711 (99.6%) were on DTG-containing regimens, and only 2 (0.3%) were on a regimen with a ritonavir-boosted protease inhibitor. Most participants (686 [96.6%]) were virally suppressed. Treatment for comorbidities was common, with 175 (24.5%) on treatment for hypertension and 39 (5.5%) on treatment for diabetes mellitus. No participant reported current use of traditional medicines or non-steroidal anti-inflammatory drugs. The median eGFR was 64.7 mL/min/1.73m^2^, and 289 (40.5%) participants had an eGFR < 60 mL/min/1.73m^2^. The margin of error for the prevalence of eGFR < 60 mL/min/1.73m^2^ among the study population is +/- 3.60% with a 95% confidence level. When adjusting for the predicted effect of DTG on eGFR, the median eGFR was 71.9 mL/min/1.73m^2^ and 200 (28.0%) participants had an eGFR < 60 mL/min/1.73m^2^.

**Table 1:** Characteristics of HIV-positive elderly adults (≥ 60 years old) on antiretroviral therapy receiving outpatient HIV care in Kenya.

Participant Characteristic	Total (n = 714)
Age (years)*	64.0 (60.0, 87.0)
Gender	
Female	386 (54.1%)
Male	328 (45.9%)
Race: Black	714 (100%)
On treatment for hypertension	175 (24.5%)
On treatment for diabetes mellitus	39 (5.5%)
HBV co-infection	16 (2.3%)
Median nadir CD4 count (cells/μL)	246 (114, 394)
Category nadir CD4 count (cells/μL)	
< 50	77 (11.7%)
50–199	196 (29.7%)
200–349	183 (27.8%)
350–499	112 (17.0%)
≥ 500	91 (13.8%)
HIV-1 viral load < 50 copies/mL	686 (96.6%)
Time since HIV diagnosis (years)	13.1 (8.9, 15.7)
Time on ART (years)	9.5 (7.1, 9.7)
Current ART regimen	
DTG/TDF/3TC	663 (92.9%)
DTG+ABC/3TC	46 (6.4%)
DTG+AZT/3TC	2 (0.3%)
EFV/TDF/3TC	1 (0.1%)
ATV/r+TDF/3TC	1 (0.1%)
LPV/r+TDF/3TC	1 (0.1%)
TDF exposure (ever)	702 (98.3%)
Time on TDF (years)	8.0 (0.9, 15.8)
eGFR (mL/min/1.73m^2^)	64.7 (52.0, 78.0)
eGFR (mL/min/1.73m^2^) with adjustment for DTG**	71.9 (57.8, 86.7)
eGFR category	
≥ 90 mL/min/1.73m^2^	78 (10.9%)
60 to 89 mL/min/1.73m^2^	347 (48.6%)
30 to 59 mL/min/1.73m^2^	278 (38.9%)
< 30 mL/min/1.73m^2^	11 (1.5%)
eGFR category with adjustment for DTG**	
≥ 90 mL/min/1.73m^2^	140 (19.6%)
60 to 89 mL/min/1.73m^2^	374 (52.4%)
30 to 59 mL/min/1.73m^2^	193 (27.0%)
< 30 mL/min/1.73m^2^	7 (1.0%)

Data are median (IQR) or n (%) unless otherwise stated. Abbreviations: 3TC, lamivudine; ABC, abacavir; ARVs, antiretroviral drugs; ATV/r, ritonavir-boosted atazanavir; AZT, zidovudine; eGFR, estimated glomerular filtration rate using Chronic Kidney Disease Epidemiology Collaboration equation 2021 refit without race; DTG, dolutegravir; EFV, efavirenz; HBV, hepatitis B virus; HIV-1, human immunodeficiency virus type 1; LPV/r, ritonavir-boosted lopinavir; TDF, tenofovir disoproxil fumarate.

*Data are median (min, max).

**Adjustment for predicted effect of DTG on eGFR was performed by dividing calculated eGFR by 0.900

In multivariate analysis, factors associated with lower eGFR were female gender (p<0.001), being on treatment for hypertension (p<0.001) and nadir CD4 count < 50 cells/μL (p = 0.008) (Table 2).

**Table 2:** Univariate and multivariate regression analysis of factors associated with decreased kidney function among HIV-positive elderly adults (≥ 60 years old) on antiretroviral therapy receiving outpatient HIV care in Kenya.

Variable	Univariate coefficient (95% CI)	Univariate p-value	Multivariate coefficient (95% CI)	Multivariate p-value
**Age (years)**	-0.16 (-0.45, 0.14)	0.294	-0.19 (-0.50, 0.12)	0.239
**Gender**				
Female	Ref		Ref	
Male	7.36 (4.80, 9.91)	< 0.001	7.88 (5.14, 10.63)	< 0.001
**On treatment for hypertension**	-6.48 (-9.47, -3.5)	< 0.001	-6.07 (-9.18, -2.95)	< 0.001
**On treatment for diabetes mellitus**	-2.57 (-8.29, 3.15)	0.378		
**HBV co-infection**	-0.92 (-9.71, 7.88)	0.838		
**Category nadir CD4 count (cells/μL)**				
200–349	Ref		Ref	
< 50	-4.13 (-8.84, 0.59)	0.086	-6.44 (-11.17, -1.72)	0.008
50–199	-1.52 (-5.09, 2.05)	0.402	-3.13 (-6.7, 0.44)	0.086
350–499	1.02 (-3.15, 5.19)	0.631	-0.69 (-4.82, 3.44)	0.743
≥ 500	-3.49 (-7.95, 0.96)	0.124	-2.75 (-7.16, 1.66)	0.221
**HIV-1 viral load ≥ 50 copies/mL**	6.33 (-0.85, 13.52)	0.084		
**Time on TDF (years)**	-0.10 (-0.46, 0.26)	0.568	0.09 (-0.28, 0.46)	0.628

Estimated glomerular filtration rate was calculated using Chronic Kidney Disease Epidemiology Collaboration equation 2021 refit without race. Abbreviations: ARVs, antiretroviral drugs; HBV, hepatitis B virus; HIV-1, human immunodeficiency virus type 1; TDF, tenofovir disoproxil fumarate.

The median eGFR with the MDRD race-neutral equation was 57.8 mL/min/1.73m^2^ (difference compared to CKD-EPI 2021: -10.6%; correlation co-efficient 0.81) and median CrCl with CG equation was 61.5 mL/min (difference compared to CKD-EPI 2021: -4.9%; correlation co-efficient 0.68), Table 3.

**Table 3:** Comparison of creatinine-based estimating equations of kidney function among HIV-positive elderly adults (≥ 60 years old) on antiretroviral therapy receiving outpatient HIV care in Kenya.

	Median (IQR)	Correlation coefficient
**CKD-EPI 2021 eGFR (mL/min/1.73m** ^**2**^ **)**	64.7 (52.0, 78.1)	Ref
**MDRD eGFR (mL/min/1.73m** ^**2**^ **)**	57.8 (47.0, 69.5)	0.81
**CG CrCl (mL/min)**	61.5 (50.7, 76.0)	0.68

Abbreviations: CKD-EPI 2021, Chronic Kidney Disease Epidemiology Collaboration equation 2021 without race; CG, Cockcroft Gault equation; CrCl, creatinine clearance; eGFR, estimated glomerular filtration rate; MDRD, Modification of Diet in Renal Disease study equation without race.

## Discussion

In this cross-sectional study among elderly PLWH on ARV therapy receiving outpatient HIV care in Kenya, we found that 40.5% of participants had an eGFR < 60 mL/min/1.73m^2^ (or 28.0% with eGFR < 60 mL/min/1.73m^2^ after adjusting for the predicted effect of DTG on eGFR).

We found a higher prevalence of impaired kidney function than other studies among PLWH in East Africa [18], which is likely explained by the more advanced age of our study population, as well as the presence of other known risk factors, including 93.3% of participants on TDF-containing regimens, 24.5% on treatment for hypertension and 5.5% on treatment for diabetes mellitus. Although there is a lack of data on kidney dysfunction in older PLWH in Kenya or East Africa, results from the General Population Cohort in Uganda (9.7% of whom were HIV positive) found eGFR < 60 mL/min/1.73m^2^ in 2.8% of the 55–64 year-old stratum, 6.5% of the 65–74 year-old stratum, and 14.4% of the 75 years and greater stratum using CKD-EPI, which are lower than our findings for elderly PLWH [22].

These study results have important implications for the routine management of the aging population of PLWH in Kenya and potentially beyond. Routine screening for kidney dysfunction is a first step in identifying people with CKD who require: evaluation for reversible causes, management to prevent or slow progression of kidney impairment, and treatment for complications of CKD [23]. Clinicians also need to be aware of kidney function when prescribing drugs that may be contraindicated based on kidney dysfunction or require dose adjustments for kidney impairment, including many ARVs, drugs for preventing and treating opportunistic infections, and drugs for managing comorbidities which are more common among elderly PLWH [23].

Given that TDF is generally not recommended for people with pre-existing kidney dysfunction [24–26], our study findings call into question the appropriateness of TDF-containing regimens among elderly PLWH in Kenya and beyond, including the current WHO-recommended preferred and alternative first-line regimens for this population [14]. In settings like Kenya where screening for kidney impairment is not widely accessible (despite being recommended), alternative regimens should be considered for ARV initiation.

A strength of our study is the inclusion of data from all participants who were screened for a clinical trial, before applying enrolment criteria. Our population is likely representative of elderly PLWH receiving outpatient HIV care in Kenya, the majority of whom are virally suppressed and currently on a regimen of DTG, TDF and lamivudine [27]. Another strength of this study is the focus on PLWH aged 60 years and above, which provides a relatively large sample of elderly participants compared to other studies.

A limitation of this study is the cross-sectional nature of these screening data and reliance on creatinine-based estimating equations for kidney function. We report a single measure of creatinine to estimate kidney function and cannot make assumptions of CKD or tease out the contribution of acute kidney injury among these patients. A recent study evaluating the performance of creatinine-based equations (CG, CKD-EPI, MDRD and a new equation developed for the study) to estimate kidney function in sub-Saharan Africa found these equations underestimated the proportion of people with GFR < 60 mL/min/1.73m^2^ by more than half [17]. Another limitation is that we have not collected samples for urine protein, which may have identified additional participants with kidney disease. As well, data on important comorbidities or potential confounders was limited; analysis of diabetes and hypertension were based on self-report of receiving treatment for the comorbidities at time of screening rather than based on diagnostic evaluations and we did not have data for calculating body-mass index. Our analysis is also limited by the effect of DTG on CrCl. We applied a correction factor to account for this; however, the correction factor is not validated and the effect size of DTG on CrCl among people with pre-existing kidney impairment is not known [20, 21]. Our adjustment may result in an over- or under-estimation of kidney impairment among the study population.

In conclusion, our study shows high rates of impaired kidney function amongst elderly PLWH in Kenya. Further detailed and longitudinal studies are needed to assess causes and progression. This study highlights the importance of routine assessment of kidney function and the need to address modifiable risk factors, use of appropriate ARV regimens, and management of kidney disease in this population.

## Supporting information

S1 File Formulae used for calculating estimates of kidney function. (DOCX)
