# Supplementary material for: High rates of kidney impairment among older people (≥ 60 years) living with HIV on first-line antiretroviral therapy at screening for a clinical trial in Kenya
Source: PLoS One. 2023 Jun 23;18(6):e0285787. doi: 10.1371/journal.pone.0285787 (PMC10289444; doi:10.1371/journal.pone.0285787)
Supplement: S1 File — (DOCX) [file pone.0285787.s001.docx]

**S1 File: Formulae used for calculating estimates of kidney function**

Chronic Kidney Disease Epidemiology Collaboration equation 2021 refit without race (CKD-EPI 2021)

Estimated glomerular filtration rate (mL/min/1.73m^2^) = 142 X min(Scr/k,1)^α^ X max(Scr/k,1)^-1.200^ X 0.9938^age^ X 1.012 [if female] where Scr is serum creatinine (umol/L), k is 61.9 for females and 79.6 males, α is -0.241 for females and -0.302 for males, min indicates the minimum of Scr/k or 1, max indicates the maximum of Scr/k or 1

Modification of Diet in Renal Disease study equation without race (MDRD)

Estimated glomerular filtration rate (mL/min/1.73m^2^) = 175 × [S_cr_/88.4] ^−1.154^ × age^−0.203^ multiply by 0.742 [if female]. S_cr_ is umol/L

Cockcroft Gault equation (CG)

Estimated creatinine clearance (mL/min) = (140 – age)* (weight in kg) / (0.814*Serum creatinine (µmol/L)). For females, multiply result by 0.85
